# Food environment components influencing consumption trends of neglected and underutilized species in northern Benin

**DOI:** 10.1371/journal.pone.0314438

**Published:** 2025-09-03

**Authors:** Bissola Malikath Verena Bankole, Waliou Amoussa Hounkpatin, Sam Bodjrenou, Julia Bodecker, Lynn Julen, Flora Josiane Chadare, Celine Termote

**Affiliations:** 1 School of Nutrition, Food Science and Technology, Faculty of Agronomics Sciences, University of Abomey-Calavi, Abomey-Calavi, Benin Republic; 2 Alliance Bioversity International and CIAT, Abomey-Calavi, Benin Republic; 3 Alliance Bioversity International and CIAT, Nairobi, Kenya; 4 University of Natural Resources and Life Sciences, Vienna, Austria; 5 Laboratory of Science and Technology of Food and Bioresources and Human Nutrition, Sakété University Center, National University of Agriculture, Sakété, Benin Republic; Kerman University of Medical Sciences, IRAN, ISLAMIC REPUBLIC OF

## Abstract

Malnutrition is a global problem driven by food systems that impact climate and biodiversity. Neglected and underutilized species (NUS) could improve diets, but what drives their choices and consumption, particularly in low and middle-income countries, is poorly documented. This study investigated the influences of the personal food environment on the consumption of NUS in three communities in the department of Atacora in northern Benin. Following a purposive sampling strategy, 24 semi-structured interviews were conducted with locals and 18 with professional experts. 12 group discussions with villagers from six villages in the three communes complemented and deepened the information gathered, focusing on both barriers and factors for enhancing consumption. The data collected was translated and transcribed into French and analyzed using qualitative content analysis with Atlas.ti software. Among the investigated plant parts, an upward trend was found for *Moringa oleifera* leaves and *Vigna radiata* seeds, a downward trend for *Adansonia digitata* pulps, and a varied trend for *Ocimum gratissimum* leaves and *Adansonia digitata* kernels and leaves. Drivers for changes in consumption were found in all four dimensions of the food environment. Among the plant parts with the main increasing trend in consumption, various aspects of desirability, above all increased food and nutrition knowledge and skills, led to a positive consumption trend. The downward trend has most often been attributed to declining accessibility, but several aspects have also made these plant parts less affordable and less desirable (taboos for example). The strong variation in dimensions’ influence on plant parts with variable trends reflects their non-unanimous changes. Research and policies should address the factors influencing the consumption of these foods. Making neglected species more accessible, affordable, and desirable can enhance food security and environmental sustainability.

## 1. Introduction

Six years from the 2030 deadline, hunger and food insecurity trends remain off track to end hunger and food insecurity by 2030 [1]. Progress indicators towards global nutrition goals also show that the world is not on the right track to eliminate all forms of malnutrition. According to FAO *et al.* [2], approximately 2.4 billion people will still lack access to nutritious, safe, and sufficient food in 2022. Rapid population expansion, natural deterioration, and urbanization make it difficult for developing countries to ensure food security [3]. A sustainable food system is defined as one that guarantees food security and nutrition for all so that the economic, social, and environmental bases for generating food security and adequate nutrition are not compromised for future generations [4]. Today’s food systems, dominated by a few highly uniform crops, are detrimental to our nutrition, health, and environment, as well as to the cultural identity of populations, their autonomy, and the economic empowerment of communities engaged in agriculture and vulnerable groups [5]. Due to the serious extinction of the genetic heritage caused by agricultural modernity, most species are on the point of extinction and are therefore considered neglected. Since most species are still unknown, even though they are capable of providing a secure food supply, maintaining genetic variety has proved difficult [3]. Of the 90% of undiscovered plant species, 77% are threatened with extinction, and the severity is extreme for the African continent, where extinction could reach 45% by 2085, and 97% would observe a reduction in plant species diversity [6]. In light of the key targets of SDG 2, “Zero Hunger”, which seeks to promote human nutrition, ensure food security, and end hunger, the effective use of neglected nutrient-dense domestic, wild, regional, semi-domesticated, traditional, and niche species could remedy these problems.

Indeed, Neglected and underutilized species (NUS) encompass wild, semi-domesticated, or fully domesticated plants in various forms, such as field crops, trees, shrubs, and vines, along with edible fungi and animal species whose potential is not fully exploited [7]. Many NUS have similar or better nutritional profiles than most foods [5]. Given the quantity of minerals, vitamins, and carotenoids that neglected and underutilized species (NUS) provide, their contribution to improving the micronutrient content of the human diet could be significant [3]. Profitability is driven by NUS cultivation due to low input demand and resilient farming practices [8]. Although most small-scale growers face poor production and incomes, these economically important species can be used to benefit disadvantaged farmers or growers. Seventeen sustainable development projects are to be completed by 2030, the second goal being a world without hunger, the main challenge remaining food security [9]. The efficient use of neglected nutrient-rich domestic, wild, regional, semi-domesticated, traditional, and niche species could remedy these problems [10]. Consequently, the identification of potential natural resources, including plant resources, has become vital. NUS are also recognized as a promising solution to the problems of nutrition and hunger in the context of climate change [11]. Despite their potential, various geographical, agronomic, economic, and socio-cultural aspects in food system value chains represent barriers to NUS consumption [7,8]. More specifically, these barriers are mainly attributed to the poor economic competitiveness of NUS compared to staple crops, inefficiencies in the production, storage, and processing of NUS, the lack of sound baseline data on the nutritional and protective properties of NUS, disorganized or nonexistent food supply chains, negative associations with a poor rural lifestyle and low social status [8]. Despite the recognized nutritional potential of neglected and underutilized species (NUS), their integration into household diets remains limited due to measurable economic constraints particularly pricing disparities between NUS and conventional staple crops. For example, Adansonia digitata pulp is often diverted to commercial markets due to its high economic value, reducing its availability for home consumption. This economic trade-off has significant nutritional implications, especially in regions where child stunting rates exceed 30% [12,13] and iron-deficiency anemia affects over 50% of women of reproductive age [14]. Quantifying and contextualizing these pricing barriers are therefore critical for understanding how they exacerbate nutritional vulnerability in food-insecure populations. Thus, food choice and consumption are influenced by various physical, economic, social, and cultural factors [9] and are dynamic, contextual, multifaceted, and multilevel [10].

**Fig 1:**
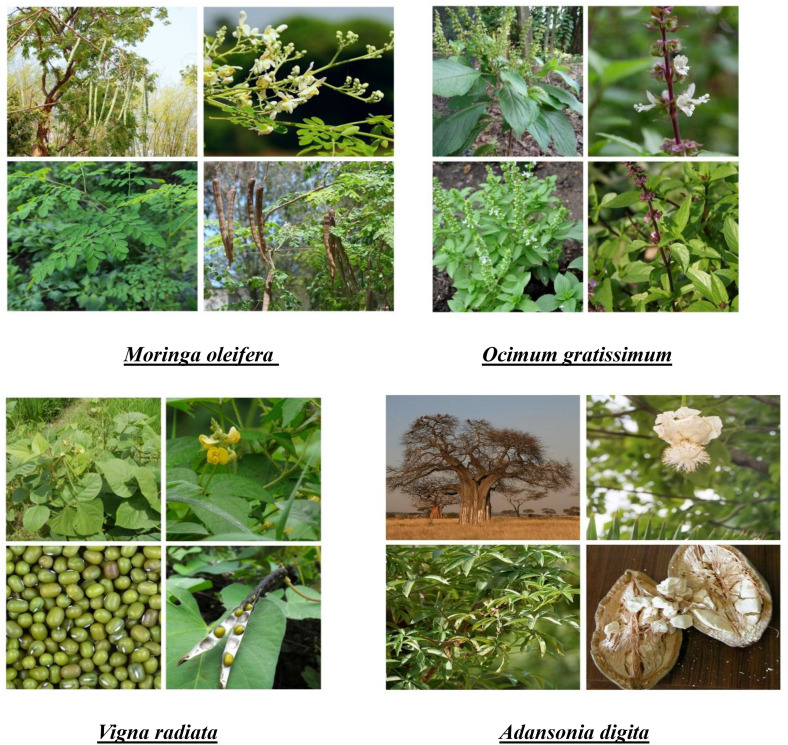
Neglected and underutilized species targeted.

Food environments serve as the interface for individuals to interact with the larger food system for food acquisition and consumption [11]. The internal domain consists of individual-level dimensions of accessibility, affordability, convenience, and desirability, whereas the external domain includes availability, prices, vendor and product properties, and marketing and regulation [15]. In the rural food environments of low-income countries, food consumers are often food producers, and many foods are produced and obtained as part of the local food system [16]. Their food environment is inherently connected to the local production system and the well-being of the local ecosystem. Thus, food is often acquired through the natural and built food environment, including market acquisition, crop harvesting, and wild collection, as well as through non-monetary transfers [17,18]. Reintroducing NUS into food environments could improve access to and utilization of nutritious foods, leading to healthier diets [5]. There is a growing need to improve the use of neglected and underutilized species (NUS), which are undervalued in current food environments, despite being often highly nutritious and resilient to climate change, and could offer new income-generating opportunities [19]. A better understanding of local food environments at NUS, including the drivers of consumption, is therefore essential to inform subsequent nutrition interventions aimed at improving food and nutrition security, environmental security, and strengthening livelihoods [20]. Thus, this study aimed to identify the personal food environment factors influencing the consumption of four targeted plant species (*Adansonia digitata, Moringa oleifera, Ocimum gratissimum and Vigna radiata*) (Fig 1) by people living in three (03) of the seven (07) communes in the department with high levels of food insecurity: Natitingou with 27.8%, Tanguiéta with 26.5% and Toucoutouna with 29.8% of household’s global food insecure [21].

## 2. Materials and methods

### 2.1. Sampling

In each commune, two villages were randomly selected, namely Kampouya and Koukouarbirgou in the commune of Natitingou, Tectibayaou and Wabou in the commune of Toucountouna, and Douani and Kosso in the commune of Tanguiéta. Purposive sampling [22] was used in this study to select informative interview partners in the area of interest. The actors to be included in each key informant category were identified by recommendation. Saturation theory was used to determine the sample size. Two groups of key informants were identified based on the following required information on the personal food environment and its influences on the consumption of the selected NUS. The first group was made up of local experts who were chosen based on the following criteria:

- People who are at least 50 years old
- People who have lived at least 25 years in their place of residence within the study zone (six villages of the communes of Natitingou, Toucountouna, and Tanguiéta) and who have experienced changes that took place over the last decades about the plant species consumption.

The second group of key informants consisted of professional experts who were selected based on the following criteria:

- Stakeholders who work in agriculture, nutrition, and health in the study zone (communes of Natitingou, Toucountouna, and Tanguiéta) and are in direct contact with the local population through their working activities. They can help explain people’s food choices.
- Preferably, people who work on different levels (villages, communes, etc.) to obtain a multi-level perspective

Focus groups were also held with people of different ages, as well as religious, educational, and sociolinguistic groups, to validate and complement the information obtained from the semi-structured interviews. Participants in the semi-structured interviews with local experts and village chiefs were excluded as their opinions had already been taken into account, and more complementary data is now hoped for. Preferably, no participants from the same household were selected. Discussions with men and women were conducted separately to enhance the free exchange of opinions and ensure the homogeneity of the focus groups. For preference, people representing the residents of the villages and therefore coming from various age groups (+18 years and above) and religious, ethnic, educational, etc., backgrounds. Then, respectively, 24 local experts and 18 professional experts were selected for this study, while 12 focus groups were done. However, information was collected until saturation was reached.

### 2.2. Ethics approval and consent to participate

This study was conducted according to the guidelines laid down in the Declaration of Helsinki. Then, the study protocol and all procedures involving research study participants were approved by the Benin National Health Research Ethics Committee (CNERS-www.ethique-sante.org) with ethical approval number No.46 of November 07^th,^ 2019, and reference number No_093/MS/DC/SGM/DRFMT/CNERS/SA. Before each interview or group discussion, all participants gave written informed consent after receiving the project’s background and interview procedure in the local language.

### 2.3. Data collection

This study was conducted from November 10, 2019, to December 10, 2019. A pre-test was carried out to improve the different questionnaires. All participants gave written informed consent after receiving full information about the study in the local language. The first phase consisted of semi-structured interviews with local and professional experts from different perspectives on the personal food environment. Local experts were questioned about their personally perceived changes in the consumption of the selected NUS and related changes in their food environment, while professional experts were asked about the changes they perceived while working with local community members within the research area. Then, for each NUS selected, an in-depth discussion was held on questions relating to all dimensions of the personal food environment.

Focus group discussions (FGD) took place in the second step. Questions were asked about the dimensions and aspects of food environments related to the four selected NUS. Examples of positive and negative influences of the factors drawn from the semi-structured interviews were integrated into the focus group questionnaire to deepen their understanding. During data collection, the same visual stimuli were used in the form of photographs of the selected NUS to make them easily identifiable. Photographs were numbered from 1 to 4 to facilitate handling of the different species (particularly for ranking tasks).

### 2.4. Data management and analysis

A total of 24 semi-structured interviews with local experts and 12 focus group discussions were recorded in local languages. All the recordings have been fully translated and transcribed into French.

#### 2.4.1. Ranking.

Data saved were analyzed from a content perspective. Quantitative data from the rankings that originate from ordering photographs of the four NUS according to specific criteria were analyzed by descriptive statistics (frequencies). The scores cited by each respondent were added for each plant, resulting in a total score for each NUS. In the second step, this total is divided by the number of interview partners, which resulted in a mean for each NUS. The lower the mean value, the higher was the rank of the species for the specific criterion [23]. The overall mean rankings for the four species were then calculated from the ranked sums of the different interview groups (local and professional experts as well as focus groups).

#### 2.4.2. Qualitative content analysis.

Content structuring aimed to systematically extract and summarize specific aspects from the coded material, which were primarily determined by the deductively derived category system [24]. Thus, the changes in consumption and the related drivers of change, as well as contemporary barriers and enhancing factors of consumption, were displayed in a more comprehensive form. The first step of the qualitative content analysis involved coding, where relevant information from the 54 transcription documents was extracted according to the developed categories. The second step of content structuring consisted of paraphrasing, generalizing, and reducing the coded text passages and finally led to derived answers to the research questions. All text material was coded after the same procedure using the software ATLAS. ti version 7.5.18 (Scientific Software Development GmbH, Berlin, Germany).

#### 2.4.3. Coding.

The unit of analysis consisted of 42 semi-structured interviews and 12 focus group discussions. Then, for each of the four (04) NUS, 13 distinct categories were created (Table 1). Categories 1–8 included relevant dimensions and aspects derived from the conceptual framework. Category 9, called “additional factors”, included relevant information influencing the consumption of the corresponding species, which could not be classified in one of the other existing categories. In addition, categories 10–13 described positive, negative, and neutral changes in the consumption of each species, as well as changes in consumption patterns over time and in the present. Initially, the material was systematically filtered for information based on the category system. The unit of analysis was defined as the smallest possible but still comprehensible passage of text. Information relating to the characteristics of a specific code was extracted, even in the absence of direct reference to the factor’s influence on consumption (increase/decrease/neutral change/change). If there was a direct link between factors and their effects on consumption, the text passage was coded with the corresponding factor and the effect on consumption (positive, negative, neutral effect, or change in consumption pattern). In addition, text passages were classified into several categories [1–9] if the information they contained referred to more than one category. In the group discussions, the coded text contained statements from one or more people. Some of the additional factors were added to one of the existing categories as they were identified as underlying drivers. Indeed, the hierarchical coding system used in this study was informed by and aligned with broader frameworks in food systems modelling and behavioral economics. Specifically, the categorization of personal food environment dimensions such as accessibility, affordability, convenience, and desirability draws directly from the global conceptual model proposed by Turner et al. [25], which distinguishes between internal and external food environment domains. This structure also reflects principles from economic utility theory, where food choices are shaped by opportunity costs, budget constraints, and time allocation [10]. Additionally, the inclusion of categories such as social markers and informal institutions speaks to the integration of behavioral economics, acknowledging that consumer choices are influenced by social norms, cultural identity, and perceived status.

**Table 1:** Category developed.

Number	Code	Description
Personal food environment dimensions		
1	Accessibility	Physical distance, time- and transport-based aspects relative to individuals that influence consumption of the species X
2	Affordability	Purchasing power which influences consumption of the species X
3	Convenience	Time and effort needed to influence consumption of the species X
4	Informal institutions	Norms of behaviors, conventions, and self-imposed codes of conduct that influence consumption of the species X
5	Social markers	Social status and group membership that influence the consumption of species X
6	Ecological and biological knowledge and skills	Knowledge of phenology, propagation modes, and seed storage methods and about the relationship between humans, animals, plants, and the physical elements of their environment as well as related management, which influence consumption of the species X
7	Food and nutrition knowledge and skills	Attributes related to facts and information acquired through experience or education related to foods and nutrition as well as techniques of food purchasing, preparation, handling, and storage which influence consumption of the species X
8	Preferences for sensory properties	Sensory responses to taste, smell, consistency, texture, and visual appeal that influence consumption of the species X
Additional factors		
9	Additional factors	Factors that cannot be categorized into one of the categories and which influence consumption of the species X
Changes in the consumption of the species		
10	Increase of consumption level	Positive change of consumption level of the species X
11	Decrease of consumption level	Negative change of consumption level of the species X
12	Neutral change of consumption level	No change of the consumption level of the species X
13	Change of consumption mode	Change in the way of consumption of the species X

#### 2.4.4. In-depth analysis and content structuring.

As a first step, the coded text material was translated and paraphrased from French into English and generalized according to a defined level of synthesis. To this end, the text passages coded in the thirteen categories were extracted from the ATLAS. ti software into separate Excel files. Each Excel file contained a separate table sheet for each of the 4 NUS. The level of generalization of the synthesis was defined as the set of characteristics with direct or indirect statements on their influence on the consumption of specific plant parts and/or on the evolution of plant consumption over time. After paraphrasing and generalizing all statements, reduction encompasses the review of all generalized statements and is divided into two sub-steps. The statements with redundant information were reduced, whereas the whole variation in information was kept. Firstly, the matrices resulting from the first reduction step included information on consumption trends and past and current consumption levels for each plant species or part studied. For that, the information from codes 10–13 (changes in the consumption of the species) was used and was quantitatively analyzed. Then, the respective statements were counted separately for semi-structured interviews and focus group discussions. The statements about increased/decreased/unchanged consumption were counted per focus group (unlike in semi-structured interviews where they were counted per person). Repeating statements by the same interview partner/focus group were counted once. A net change was calculated for each group by subtracting the number of declarations of decreased consumption from the number of declarations of increased consumption and dividing by the total number of declarations. The total number of statements was the sum of all the declarations obtained for the plant part concerned. The overall net change for consumption of each investigated plant part resulted from the difference of the total number of increased minus decreased statements divided by the total number of statements:


$$Overallnetchange=\frac{\text{Total number of increased statements}-\text{decreased statements}}{\text{Total number of statements}}$$

These numbers varying from +1 to −1 were classified into three main trends:

- increasing trend in consumption (1 to 0.33)
- varying trends in consumption (0.33 to – 0.33)
- negative trend in consumption (−0.33 to −1)

Then, information from the matrices of codes 1–9 was used to explain how the dimensions of the food environment can explain the different trends obtained for the different parts of the species consumed.

## 3. Results

### 3.1. Participant characteristics

All the local experts were farmers (100%), and the average age was 59 years (Table 2). The average duration they had lived in the research villages was 57 years. The interviewees belonged to the Waama (62.5%), Natimba (29.1%), and Otamari (8.3%) ethnic groups. Around half of those interviewed (54.1%) had received no formal education, while the others had attended elementary school. As the professional experts, more than half were men (66.6%) aged between 24 and 52 years. Most of them (61.1%) worked as nutritional advisors, followed by agricultural advisors (22.2%) and the others were social workers and scientists. The professional experts belonged to diverse ethnic groups. About 66.6% of the professional experts had a bachelor’s degree. Regarding the focus group, the respondents’ mean age was 41 years, ranging from 18–80 years. The mean residential duration in the research villages was 35 years, ranging between 6–72 years. All respondents were farmers. Religious affiliation and education level varied among participants but most of them were from Waama ethnic group.

**Table 2:** Sociodemographic characteristics of participants.

Characteristics	Local experts n = 24	Professional experts n = 18	Focus group participants n = 72
	n (%)	n (%)	n (%)
**Sex**			
* Female*	7 (29.16)	6 (33.33)	36 (50)
* Male*	17 (70.83)	12 (66.66)	36 (50)
**Age (*years*)**			
* Mean*	59	34	41
* Range*	50-71	24-52	18-80
**Duration of residence (*years*)**			
* Mean*	57	N/A	35
* Range*	25-71	N/A	6-72
**Sociolinguistic group**			
* Berba*	–	3 (16.6)	2 (2.77)
* Dendi*	–	2 (11.11)	–
* Ditamari*	2 (8.33)	2 (11.11)	12 (16.66)
* Fon*	–	2 (11.11)	–
* Fulani*	–	–	1 (1.38)
* Natimba*	7 (29.16)	–	9 (12.5)
* Waama*	15 (62.5)	3 (16.66)	48 (66.66)
* Other*	–	6 (33.33)	–
**Formal instruction**			
* None*	13 (54.16)	–	33 (45.83)
*Primary school*	11 (45.83)	–	27 (37.5)
*Secondary school*	–	6 (33.33)	12 (16.66)
*University*	–	12 (66.66)	–
**Main Activity**			
*Agricultural advisor*	–	4 (22.22)	–
*Farmer*	24 (100)	–	72 (100)
*Nutritional advisor*	–	11 (61.11)	–
*Other*	–	3 (17)	–
**Religion**			
*Christianity*	16 (63)	N/A	N/A
*Muslims*	–	N/A	N/A
*Traditional religions*	4 (29)	N/A	N/A
*None*	4 (8)	N/A	N/A

### 3.2. Consumption of NUS selected

Deeper insights into the relative consumption frequencies and preferences among the four (04) NUS were indicated through their rankings (Table 3). The most and least frequently consumed species were identical with the most and least preferred ones. *A. digitata* and *M. oleifera* were the most frequently consumed and preferred species, whereas *V. radiata* and *O. gratissimum* were on the lowest ranks.

**Table 3:** Mean ranking for consumption preferences and frequencies.

Consumption preferences					Consumption frequencies				
Mean ranking		Overall mean ranking			Mean ranking		Overall mean ranking		
Local experts (n = 14)	Professional experts (n = 17)				Local experts (n = 14)	Professional experts (n = 17)			
4.00	2.35	*A. digitata*	3.18	**1** ^**st**^	3.17	2.71	*A. digitata*	2.99	**1** ^**st**^
6.29	3.71	*M. oleifera*	5	**2** ^**nd**^	6.04	3.06	*M.oleifera*	4.55	**2** ^**nd**^
4.75	6.12	*V. radiata*	5.44	**3** ^**rd**^	5.25	6.00	*V.radiata*	5.43	**3** ^**rd**^
6.92	5.71	*O.* *gratissimum*	6.31	**4** ^**th**^	7.63	5.00	*O. gratissimum*	6.31	**4** ^**th**^

### 3.3. Changes in consumption of selected NUS

Changes in consumption over the last decades varied among the selected NUS as well as among different edible plant parts of the same species (Table 4). A major increase in consumption was predominant for *M. oleifera*’s leaves and *V. radiata*’s seeds. For *A. digitata* pulps, a downward trend in consumption was predominant. For the remaining investigated plant parts of four species (*A. digitata*’s leaves and kernels and *O. gratissimum*’s leaves), statements about their consumption varied in a more balanced way between increased, decreased, and unchanged consumption.

**Table 4:** Changes in consumption of the NUS plant parts.

Species	Plant parts consumed	Number of statements by local and professional experts (N = 42)				Number of statements by focus groups (N = 12)				N = 54	
		Changes in consumption								Overall net	Main trend
		Increase	Decrease	No change	Net change	Increase	Decrease	No change	Net change		
***M. Oleifera***	**Leaves**	20	1	1	0.86	5	0	0	1.00	0.89	Increase
***V. radiata***	**Seeds**	12	7	0	0.26	5	0	0	1.00	0.42	Increase
***O. gratissimum***	**Leaves**	4	5	2	− 0.09	2	0	0	1.00	0.08	Varied
***A. digitata***	**Leaves**	4	3	3	0.10	2	0	1	0.67	0.23	Varied
***A. digitata***	**Kernels**	1	5	2	−0.50	2	0	1	0.67	−0.18	Varied
***A. digitata***	**Pulp**	3	11	3	−0.47	1	0	1	0.50	−0.37	Decrease

The clearest trend of increasing consumption was observed for M. oleifera leaves, whose consumption was non-existent or low a few decades ago. V. radiata seeds also showed a positive trend in consumption, mainly linked to the recent period. They were traditionally known and consumed in the research area, although not all those interviewed said they had eaten the species in their youth. About ten years ago, consumption of V. radiata seeds fell to a virtually non-existent level. In recent years, there has been an increase in harvests from own production and higher levels of consumption, although some respondents still mentioned a total absence of seed consumption of the species currently. New methods of preparation include incorporating the seeds into traditional meals such as the Beninese dish “Atassi/Watché” (rice with beans) or bakery products such as cakes.

Among plant parts with a downward trend, consumption of A. digitata pulp has declined. A few decades ago, pulps were considered a sweetener and were often consumed directly by children or added to porridges.

For A. digitata leaves, positive, negative, and unchanged consumption trends were mentioned, with the level of commercialization of leaves having increased over recent decades. Others stated that consumption had only slightly increased or even decreased since the leaves were already widely consumed in the past. The consumption of A. digitata kernels has undergone similar variations. The processing of A. digitata kernels into oil represented a new consumption pattern, in addition to their use in sauces. As for O. gratissimum leaves, their consumption was non-existent or low during the youth of those surveyed. Current consumption was limited to a minority of the local population but with varying trends over time.

### 3.4. Drivers for changes in consumption of the selected NUS

Multidimensional drivers within the personal food environment were identified as factors that contributed to the consumption trend of NUS.

#### 3.4.1. Moringa Oleifera.

Regarding the **accessibility dimension**, although some of those interviewed perceived no change in the accessibility of *M. oleifera,* as trees were already present nearby, growing in fields and around houses two decades ago, some changes have been noted: Indeed, improved accessibility has been achieved by increasing or emerging clean cultivation practices. *M. oleifera*, for example, started to be grown in home gardens. “*This species is produced around habitats and therefore facilitates easy access and increased consumption”* (a resident of the village of Kampouya). An additional reason for the increase in *M. oleifera* consumption is the reduction in accessible alternative food sources. There are very few vegetables accessible in the dry season apart from *M. oleifera* and *okra* (*Abelmoschus esculentus)*. In addition, we noticed temporal aspects that enhanced *M. oleifera* consumption since storage and processing extended the consumption period to the whole year.

Regarding the **affordability and convenience dimension**, sufficient quantities of the leaves were easily found in the natural environment, sometimes from the free transfers that made them affordable and convenient. On the other hand, the small size of the leaves made them easy to collect and cook.

For the **desirability dimension**, a **better awareness of the usefulness**, mainly about health and nutrition, as well as new processing, storage, and preparation techniques, have been declared. Although this species has long existed in the research area, information on the edibility and usefulness of its leaves was unknown during most of the participants’ youth. Fresh or dried leaves were used to treat various illnesses and problems (malaria, tuberculosis, AIDS, stomach pains, breastfeeding problems in women, and malnutrition). Some interviewees were aware of the edibility of the leaves but consumed them to a lesser extent than today: *Ten years ago, no, I’d say 15 years ago, I had Moringa in my neighborhood and even in my house. But we didn’t eat the leaves. So, people from Niger and Mali came to my house, always asking for the leaves. But today, I eat a lot of it and even have some at home. It’s become so much more popular than before, as people have discovered its many uses and virtues* (a resident of the village of Kampouya). Then, leaves were partially used as animal fodder during the interviewees’ youth. Information on the nutritional and health benefits of *M. oleifera* leaves has been reinforced by the promotional activities of various stakeholders, including local projects, hospitals, scientists, start-ups, and the media. These activities have raised local awareness of the leaves’ dietary benefits by recommending them as part of a recovery diet for malnourished children, by marketing products made from the species’ leaves or through information campaigns; the storage of the plant’s leaves in powder form has helped to increase consumption

Another aspect of desirability, such as **preferences for sensory properties,** contributed to the positive evolution of consumption. For example, *M. oleifera* became increasingly recognized and known as an edible species, and the formerly wild species was increasingly cultivated. Furthermore, the **social marker** aspect had a positive influence on the upward trend of *M. oleifera* consumption since their image changed from a foreign element to a more familiar and appreciated element of the human diet.

#### 3.4.2. Adansonia digitata.

A fluctuation trend can be observed for *A. digitata* parts (a decreasing trend for the pulp and a varying trend for the kernels and leaves). For the participants in this study, the decreasing trend of *A. digitata* pulp could be explained by several dimensions of the personal food environment. Their **low accessibility** was due to insufficient reproduction efforts by humans combined with a decreased productivity of elder trees due to the long lead time required to start harvesting fruit (*A. digitata*). This perennial species was destroyed by cutting or bushfires. In addition, the increased production of a variety of alternative food sources has reduced the consumption of *A. digitata* pulps. Indeed, the population depended less on the wild harvest of these fruits as they substituted them with the production of other plant species such as *Dioscorea L.* (yams), *Oryza sativa L.* (rice), and *Glycine max (L.)* (soja).

The **affordability dimension** in terms of purchasing power partially impeded or reduced the purchase and consumption of *A. digitata* pulp. Indeed, baobab pulp is one of the edible plants with a high economic value. It is therefore marketed, and the cash income from sales is used to buy food (e.g., staples, condiments) and other items (e.g., soap, washing powder, shoes, clothes). As for the leaves and kernels, for example, the increasing sales have harmed auto consumption by the population.

In terms of the **convenience dimension**, the acquisition of the pulp is a factor impeding consumption due to the unpractical harvesting, essentially linked to the high height of the baobab trees: “*Can you climb the baobab that easily? No, you can’t, unless it’s a young baobab. Then you’ll have to wait for the rainy season to start, with the high winds, before you can expect the fruit to fall”* (a resident of *Tectibayaou*’s village). Although people were aware of how to **process and store** baobab leaves throughout the year, there were still problems with hygiene standards and preserving the nutritional value of the leaves. For example, clean drying areas were often insufficient, yellow leaves were often not removed, and drying often proceeded in the sun. There is also a lack of material inputs (e.g., refrigerator, clean water, bottles) to process and store *A. digitata* pulp.

Regarding the **desirability dimension**, **informal institutions** in terms of decreased **social access** regarding harvest and transfers contributed to the downward trend of *A. digitata*’s pulps. In the past, *A. digitata* fruit that fell to the ground could be harvested by anyone, but it is becoming increasingly common for the tree’s owner to restrict access. In addition, a loss of familiarity and changes in dietary habits were contributing to the decline in consumption. For instance, mothers and grandmothers used to prepare porridges with the pulp, which nowadays has stopped. Certain **taboos** in the *Berba* and *Waama* ethnic groups impede the consumption of baobab. According to them, anyone who planted an *A. digitata* tree and consumed its fruit, leaves, or kernels was condemned to die. The reproduction of the trees was perceived as being reserved for God, even if people were allowed to maintain existing trees: “*You know, some baobabs are possessed by spirits, souls. Some old people can even feel these spirits. In such cases, they forbid their consumption at the risk of death”* (a resident of Natitingou’s village). The absence or limited reproduction of perennial species, as well as the **lack of knowledge** of their reproduction methods and the mistaken belief that these species only reproduce naturally, could lead to a reduction in consumption. Furthermore, a decrease in **sensory preferences** related to unfavorable taste perceptions such as sourness of *A. digitata*’s pulp or bitterness of old leaves.

#### 3.4.3. Vigna radiata.

Essentially, it was **dimensions of convenience** and **desirability** that had positive influences on the upward trend of *V. radiata* seeds. A few years ago, with the arrival of the variety, convenience became greater due to its shorter production cycle and shorter cooking time: “*If the first rains fall and you plant the species, it will produce about two months later. If you wish, you can plant a second seedling during the same season”* (a resident of Kosso’s village); “I’ve *been producing the species for 3 years, it’s only this year that I haven’t been able to because of my pregnancy. I love its smell and taste”* (a resident of Douani’s village). Most participants who recognized this species said it tasted very good and was much appreciated. Moreover, various known techniques for preparing *V. radiata* seeds, which were similar to other legumes such as *V. subterranea* (bambara peanut) or *V. uniguculata* (cowpea), increased their consumption “In *our cooking demonstrations, we used mung bean instead of cowpea to enhance meals. We use it to make fritters, cakes and even Watché (Rice* *+* *Beans)*” (an agent working for Giz/Prosar).

#### 3.4.4. Ocimum gratissimum.

A predominant trend of consumption of *O. gratissimum*’s leaves was marked by variation of the consumption. In fact, for **physical accessibility**, herbs like *O. gratissimum* were accessible through their wild and cultivated growth around houses. Its seeds are purposively thrown away in the fields after the harvest of their leaves to enhance their natural reproduction. *O. gratissimum* mainly grew in humid locations. Watering of women with kitchen water at drier locations increased its production and consumption. The increasing consumption was also due to its **affordable** option compared to some vegetables, which can be acquired expensive. Its preparation was found to be highly convenient due to the short amount of time needed. Positive **social maker** (**high appreciation)** of the leaves referred to their importance in dietary habits/as part of the local culture.

Then, **awareness of the health benefits** of the consumption of plant parts showed positive effects on the consumption. Indeed, the leaves have antibiotic effects mainly used to treat stomach-related diseases (e.g., intestinal infections, constipation, diarrhea, or ulcers). It constituted the first sauce that was given to women after giving birth because of its stimulating effect on milk production “ *Yes, Ocimum can be eaten, but it’s better known as a therapeutic food* “(a resident of Koukorbirgou’s village). In addition, the smell had the positive effect of reducing the undesired odor of other food, although, for new consumers of the species, the strong smell represented a barrier to consumption. Several obstacles have also been mentioned, leading to fluctuations in consumption over time. Indeed, the limited reproduction was explained by **a lack of knowledge** about their reproduction modes, by a lack of interest or incentives**. Lack of storage** and **processing techniques** or the willingness to find them decreased the leaves’ consumption.

## 4. Discussion

Regarding the investigated period, the results showed that not all species became less consumed by the research sample. Their consumption was not declining among all plant parts but showed upward, downward, and varying trends. The changes in consumption patterns of the investigated NUS were influenced by various factors across all aspects of the food environment. While sociocultural factors were found to drive declines in consumption in some studies predominantly [26], others identified accessibility and environmental factors as key drivers [27].

In this study, an uptrend in the consumption of *M. oleifera* leaves and V. radiata seeds has been found. *M. oleifera* is a traditional leaf vegetable that is widely distributed geographically in Benin [28]. This wide distribution has probably resulted from its adaptation to the different ecological conditions and its wide climatic tolerance. Indeed, *M. oleifera* was only introduced in Africa at the beginning of the 20^th^ century and was, therefore, not yet anchored in family and cultural practices [29]. Several people interviewed said they were not aware of the uses of moringa in their youth, unlike today. Other studies in Benin show that the local naming of *M. oleifera* in rural communities in Southern Benin can be interpreted as its use for food and medicinal purposes being relatively recent [30]. Then, the interviewees explained the positive effect of accessibility by the fact that it is growing more and more in home gardens. Agoyi *et al.* (2014) reported that the most common *M. oleifera* growing systems encountered in southern Benin were individuals scattered around farms, home gardens, and fences. It is, therefore, an easy species to find and can be harvested easily and free of charge in the wild or by free transfer. About **temporal aspects**, like *okra*, *M. oleifera* is also one of the food species accessible in seasons of food scarcity. The same was found in Ghana, where *M. oleifera* leaves and several other green leafy vegetables, such as African spinach (*Amaranthus hybridus)*, amaranth (*Amaranthus cruentus L.)*, leafy eggplant (*Solanum melongena L.*), and jute mallow (*Corchorus olitorius*) are available and accessible all year round [31]. An interesting finding in this study is the consistent impact of food and nutritional knowledge and skills uniformly contributing to the upward trend of *M. oleifera* leaves. This proves that, as in South Africa [32], several sources, such as the media, schools, and research, have communicated the importance of this plant in the diet of populations in the study area. Raising awareness of Moringa’s multiple uses throughout the community is necessary to create market demand and maximize resource utilization [33]. However, Cele & Mkhize [34] have shown that while awareness of the health benefits of NUS is growing, this does not necessarily translate into higher consumption rates. Even when aware of these benefits, farmers may prefer staple crops due to traditional dietary habits or the perception of NUS as “food for the poor”, leading to their underutilization [35]. Indeed, farmers and consumers still undervalue these crops due to entrenched eating habits or a lack of immediate economic incentives to switch from well-established farming practices to practices involving NUS. According to Mkhize et al. [36], for NUS to be more widely adopted, specific interventions are needed to address not only knowledge gaps but also practical and economic barriers to their cultivation and consumption. Recent studies have underscored key barriers and policy opportunities for promoting NUS in West Africa. In countries like Nigeria and Burkina Faso, adoption remains limited due to institutional gaps, weak seed systems, and minimal coordination across policy levels [37]. Governance interventions such as biodiversity conservation programs, targeted subsidies, and school feeding initiatives have shown potential to support NUS integration when aligned with local food systems. A holistic strategy combining conservation, commercialization, and community engagement is essential to realize the full potential of NUS in sustainable food systems [38].

Whereas some studies identified taste as the most important sociocultural reason for the increase in consumption of wild edible plants [26], positive influences of taste preferences were mentioned mostly for *V. radiata’s* seeds. A study conducted by Bankole *et al.* (2023) [39] showed that 83.33% and 82.69% of children, respectively, appreciated mung bean boiled with moringa leaves (MBML) and mung bean boiled with roasted maize flour (MBMF). Anggraeni & Rahmawaty (2022) [40] also found that the higher the addition of mung bean flour, the more panelists preferred brownies suweg formulated. This effect of sensory perception on food choice has been well documented, with taste being a determining factor in the adoption of new or less familiar foods [41]. As consumption has significantly increased over time, participants have observed that certain convenience-related factors have made the harvesting of this species challenging. Indeed, itchy hairs during harvest and the small size of the seeds make harvesting difficult. A study conducted by Sequeros *et al.* (2021) emphasized the improvement of mechanization to maintain profitability as rising labor costs depress *V. radiata* production. Market accessibility is limited due to low levels of production. In Kenya, for example, unstable markets and low market prices have been observed due to a lack of clear information on *V. radiata* prices set by traders, which do not always reflect the reality for producers [42].

Many of the causes of the decrease in accessibility of *A. digitata* pulp were similar to the findings of another study in Benin, which explained that it was due to limited regeneration caused by biotic and abiotic disturbances [43]. Then, several studies indicate that socio-economic reasons contribute to the decline of NUS [44]. Wild plants continue to represent an important part of the global food basket. While a variety of social and ecological factors are acting to reduce the use of wild foods, their importance may be set to grow as pressures on agricultural productivity increase [45]. In addition, as the natural food environment was the most important source of acquisition for the *A. digitata*, conservation of existing species on farms and in the wild, their ecosystems, and their genetic material is key. Some plant parts’ accessibility can be further enhanced through improved access to water during the dry season or the presence on markets. However, increased access to markets may also reduce their consumption [46]. The current decrease in consumption levels of *A. digitata* pulps is a result of their increasing sale. Other studies further support the high sales levels of the plant parts [47]. In contrast to the pulps, it is interesting to notice that the leaves’ consumption has not been negatively impacted by their marketing. It is worth mentioning that the leaves are among the ten most traded traditional vegetables in the Sudanian zone of Benin and nationally as well [48]. Additionally, they are also exported to other West African countries [49]. *A. digitata* pulp has also suffered from the difficulty of processing and storage. The study by Chadare *et al.* (2008) showed that in Benin, most ethnic groups mentioned the difficulty of certain processing operations, in particular, seed hulling, grinding, and sieving operations, as well as the problems of storing and preserving almonds and pulp.

Aspects such as negative beliefs and image and taboos were seen as barriers to NUS consumption [27,50]. According to the interviewees, the taboo on regenerating *A. digitata* had religious and cultural reasons. Other studies confirm the important socio-cultural role of *A. digitata* in local belief systems and for cultural identity in Benin [49,51]. Some strategies can be used to manage them and minimize their impact in some cases [52]. For example, understanding the elements associated with the consumption of certain plant parts could be studied, and attempting to reconnect them with more positive attributions may prove effective in encouraging their consumption. Reducing free transfers in the close social environment affected the consumption of different parts of *A. digitata,* which suffered from the negative side effects of their high sales level. Mayes et al.[45] findings imply that reluctance to purchase also harms the likelihood of consumption. This reluctance is due to a variety of factors, including perceived cost, lack of availability, and unfamiliarity with preparation and culinary uses (Mayes et al., 2012). Furthermore, if consumers perceive NUS as expensive or not offering sufficient value for money, they are less likely to make a purchase. Furthermore, if NUS are not readily available in local markets, or if consumers do not know how to prepare them, this may further hinder their willingness to purchase [45].

As for *O. gratissimum*’s leaves, inconvenient acquisition contributes to decreased consumption of the *O. gratissimum leaves.* Indeed, **lack of processing and storage facilities,** as well as a **lack of knowledge** about their reproduction modes, contributed to the reduction of leaf consumption. Dossoukpevi *et al.* (2012) [53] pointed out that climate change is hampering the good productivity of the leaves through traditional production methods (seed sowing, vegetative propagation), affecting their availability and consumption. Baco’s (2019) [54] study revealed that some households mentioned a lack of knowledge, organoleptic attributes, and totemic considerations to justify the non-consumption of certain leafy vegetables, including African basil (*O. gratissimum*). Similar reasons were noted in several other studies for the non-consumption of certain traditional leafy vegetables [55]. In other respects, an interesting finding in this study is the consistent impact of food and nutritional knowledge and skills uniformly contributing to the varied trend as well. A study confirms the strong link of *O. gratissimum* consumption to medicinal use awareness in terms of antibiotic properties [48]. Dansi *et al..* (2008) also showed that consumption was higher during menstruation, pregnancy, and breastfeeding. The therapeutic properties were multiple and linked to the leaves’ ability to eliminate blood clots, facilitate childbirth, eliminate waste after delivery, treat post-partum infections, heal wounds, and stimulate milk secretion. Reducing free transfers in the close social environment affected consumption of *O. gratissimum,* which suffered from negative side-effects of their high sales level.

### 4.1. Study limitations and recommendations

With regard to the first point, the research sample did not cover the overall diversity of the local population, particularly in terms of gender relations, age groups, sociolinguistic groups, religious affiliations, socio-economic realities, or degrees of urbanization. Another point is that interlinkages within drivers were not captured in their totality, even though the drivers influencing the consumption were captured. Overall, the intensity of influences and their interrelationships, including trade-offs between dimensions of the personal food environment, have not been fully considered. Theoretical and methodological refinements are necessary to measure the various types (natural/built), domains (internal/external), and parameters (dimensions and aspects) of food environments [17] for a better understanding of the factors influencing NUS consumption.

In terms of recommendations, we strongly suggest involving local communities in the promotion of neglected and underutilized species (NUS) to ensure their conservation and sustainable use. In food systems where producers and consumers intersect, involving smallholders is crucial for preserving plant species [56]. Protecting on-farm and wild species, as well as their ecosystems, is vital, given the reliance on natural environments for NUS acquisition. Enhancing access to market presence can improve the availability of specific plant parts. Educating about the nutritional benefits of these plants is essential [57]. Strengthening local social networks acknowledges their dynamic nature and fosters community cohesion. Empowering women with leadership roles and decision-making authority promotes inclusivity [58]. Strategies should address taboos and misconceptions related to plant consumption, aiming to foster positive perceptions and increase consumption.

## 5. Conclusion

The consumption of neglected and under-utilized species has a significant impact on food security through improved nutrition, greater resilience to climatic shocks, and reduced vulnerability to food price fluctuations. These species generate significant positive impacts, notably through the creation of local employment, the development of new markets, and economic empowerment particularly beneficial to women. The present study showed that among all species investigated, *A. digitata* and *M. oleifera* were the most consumed and preferred species. Consumption is not automatically declining for all species. Indeed, increased trends in consumption were found for *M. oleifera* leaves and *V. radiata* seeds, while *A. digitata* pulp consumption decreased. The results provide useful insights for developing effective entry points for improved consumption of the NUS studied by characterizing barriers and improvement factors in the various sources of acquisition and consumption processes. To address the identified barriers to NUS consumption in all four dimensions of the local food environment, strategies should focus on improving infrastructure, knowledge, and market access. This includes investing in local processing and storage facilities, offering training in reproductive techniques, and launching awareness campaigns to combat negative perceptions and taboos. More research and supportive measures in all four dimensions of the personal food environment are needed to create supportive food environments.

## References

[1] Unicef. The state of food security and nutrition in the world 2024. 2024. https://openknowledge.fao.org/items/09ed8fec-480e-4432-832c-5b56c672ed92

[2] FAO, FIDA, OMS, PAM. L’état de la sécurité alimentaire et de la nutrition dans le monde 2023. FAO; 2023. http://www.fao.org/documents/card/fr/c/cc3017fr

[3] Talucder MSA, Ruba UB, Robi M d AS. Potentiality of Neglected and Underutilized Species (NUS) as a future resilient food: a systematic review. J Agric Food Res. 2024;16:101116.

[4] HLPE FL. Waste in the Context of Sustainable Food Systems. A report by the high level panel of experts on food security and nutrition of the committee on world food security. Rome; 2014.

[5] Kennedy G, Hunter D, Garrett J, Padulosi S. Leveraging agrobiodiversity to create sustainable food systems for healthier diets. 2017. https://cgspace.cgiar.org/items/a3b96d03-8498-466a-948c-298a57c9a1b5

[6] Lenman. Lenman: On becoming extinct. 2022 [Accessed 2025 March 31]. https://scholar.google.com/scholar_lookup?title=Are%20Plant%20Species%20Also%20Becoming%20Extinct&publication_year=2024&author=I.%20Sanmart%C3%ADn

[7] Padulosi S, Hoeschle-Zeledon I. Underutilized plant species: what are they? LEISA-LEUSDEN. 2004;20:5–6.

[8] Baldermann S, Blagojević L, Frede K, Klopsch R, Neugart S, Neumann A, et al. Are neglected plants the food for the future? Crit Rev Plant Sci. 2016;35(2):106–19. doi: 10.1080/07352689.2016.1201399

[9] Powell B, Maundu P, Kuhnlein HV, Johns T. Wild foods from farm and forest in the East Usambara Mountains, Tanzania. Ecol Food Nutr. 2013;52(6):451–78. doi: 10.1080/03670244.2013.768122 24083514

[10] Sobal J, Bisogni CA, Jastran M. Food choice is multifaceted, contextual, dynamic, multilevel, integrated, and diverse. Mind Brain Educ. 2014;8(1):6–12.

[11] Bélanger J, Pilling D. The state of the world’s biodiversity for food and agriculture. Food and Agriculture Organization of the United Nations (FAO); 2019. https://www.cabdirect.org/cabdirect/abstract/20193206813

[12] Addo IY, Boadu EF, Osei Bonsu E, Boadi C, Dadzie FA. Prevalence and factors associated with undernutrition among children under the age of five years in Benin. PLoS One. 2023;18(8):e0289933. doi: 10.1371/journal.pone.0289933 37561793 PMC10414565

[13] INSAE, ICF. IEnquête Démographique et de Santé au Bénin, 2017-2018. Cotonou, Bénin et Rockville, Maryland, USA: INSAE et ICF; 2019.

[14] FAO, AUC, ECA, WFP. Africa - Regional Overview of Food Security and Nutrition 2023. 2023. http://www.fao.org/documents/card/en/c/cc8743en

[15] Turner C, Aggarwal A, Walls H, Herforth A, Drewnowski A, Coates J, et al. Concepts and critical perspectives for food environment research: a global framework with implications for action in low- and middle-income countries. Global Food Sec. 2018;18:93–101. doi: 10.1016/j.gfs.2018.08.003

[16] Raneri JE, Padulosi S, Meldrum G, King OI. Promoting neglected and underutilized species to boost nutrition in LMICs. UNSCN Nutrition. 2019.

[17] Turner C, Kalamatianou S, Drewnowski A, Kulkarni B, Kinra S, Kadiyala S. Food environment research in low- and middle-income countries: a systematic scoping review. Adv Nutrit. 2020;11(2):387–97. 31079142 10.1093/advances/nmz031 PMC7442349

[18] Dogo A, Hongbete F, Edja H, Amoussa Hounkpatin W. Characterization and challenges of food environments of children-under-five in north Benin drylands. J Agric Food Res. 2023;14:100682. doi: 10.1016/j.jafr.2023.100682

[19] Padulosi S, Thompson J, Rudebjer PG. Fighting poverty, hunger and malnutrition with neglected and underutilized species: needs, challenges and the way forward. 2013.

[20] Padulosi S, Hodgkin T, Williams JT, Haq N. Underutilized crops: trends, challenges and opportunities in the 21st century. Managing plant genetic diversity. Proceedings of an international conference, Kuala Lumpur, Malaysia, 12-16 June 2000. CABI Publishing; 2001: 323–38. doi: 10.1079/9780851995229.0323

[21] INSAE PAM. Analyse globale de la vulnérabilité et la sécurité alimentaire (AGVSA). 68. Rome, Italie: Parco de Medici; 2017.

[22] Bernard HR. Research methods in anthropology: qualitative and quantitative approaches. Rowman & Littlefield; 2017.

[23] Albuquerque UP, Cruz da Cunha LVF, de Lucena RFP, Alves RRN. Methods and Techniques in Ethnobiology and Ethnoecology. Springer New York; 2014. doi: 10.1007/978-1-4614-8636-7

[24] Mayring P, Fenzl T. Qualitative inhaltsanalyse. Handbuch Methoden der empirischen Sozialforschung. Springer Fachmedien Wiesbaden; 2019: 633–48. doi: 10.1007/978-3-658-21308-4_42

[25] Turner C, Aggarwal A, Walls H, Herforth A, Drewnowski A, Coates J, et al. Concepts and critical perspectives for food environment research: a global framework with implications for action in low- and middle-income countries. Global Food Security. 2018;18:93–101. doi: 10.1016/j.gfs.2018.08.003

[26] Thakur D, Sharma A, Uniyal SK. Why they eat, what they eat: patterns of wild edible plants consumption in a tribal area of Western Himalaya. J Ethnobiol Ethnomed. 2017;13(1):70. doi: 10.1186/s13002-017-0198-z 29233181 PMC5727875

[27] Pawera L, Khomsan A, Zuhud EAM, Hunter D, Ickowitz A, Polesny Z. Wild food plants and trends in their use: from knowledge and perceptions to drivers of change in West Sumatra, Indonesia. Foods. 2020;9(9):1240. doi: 10.3390/foods9091240 32899857 PMC7555794

[28] Dansi A, Adjatin A, Adoukonou-Sagbadja H, Faladé V, Yedomonhan H, Odou D, et al. Traditional leafy vegetables and their use in the Benin Republic. Genet Resour Crop Evol. 2008;55(8):1239–56. doi: 10.1007/s10722-008-9324-z

[29] Hedhili A, Akinyemi BE, Otunola GA, Ashie-Nikoi PA, Kulkarni M, Husson F, et al. *Moringa oleifera* Lam.: A comparative survey on consumer knowledge, usage, attitude and belief in Africa and India. South Af J Botany. 2022;147:153–62. doi: 10.1016/j.sajb.2022.01.009

[30] Agoyi EE, Assogbadjo AE, Gouwakinnou G, Okou FAY, Sinsin B. Ethnobotanical assessment of *Moringa oleifera* Lam. in Southern Benin (West Africa). Ethnobot Res App. 2014;12:551. doi: 10.17348/era.12.0.551-560

[31] Glover-Amengor M, Aryeetey R, Afari E, Nyarko A. Micronutrient composition and acceptability of *Moringa oleifera* leaf-fortified dishes by children in Ada-East district, Ghana. Food Sci Nutr. 2016;5(2):317–23. doi: 10.1002/fsn3.395 28265366 PMC5332270

[32] Fahey JW. Moringa oleifera: a review of the medical evidence for its nutritional, therapeutic, and prophylactic properties. Part 1. Trees for Life Journal. 2005;1(5):1–15.

[33] Kumssa DB, Joy EJM, Young SD, Odee DW, Ander EL, Magare C, et al. Challenges and opportunities for Moringa growers in southern Ethiopia and Kenya. PLoS One. 2017;12(11):e0187651. doi: 10.1371/journal.pone.0187651 29121079 PMC5679577

[34] Cele T, Mkhize X. Multifaceted determinants influencing South African smallholder farmers’ choices to access and utilize underutilized crops. Front Sustain Food Syst. 2025;9. doi: 10.3389/fsufs.2025.1510790

[35] Nkwonta CG, Auma CI, Gong Y. Underutilised food crops for improving food security and nutrition health in Nigeria and Uganda—a review. Front Sustain Food Syst. 2023;7.

[36] Mkhize P, Shimelis H, Mashilo J. Cucurbitacins B, E and I concentrations and relationship with drought tolerance in bottle gourd [Lagenaria siceraria (Molina) Standl.]. Plants (Basel). 2023;12(19). 10.3390/plants12193492 PMC10574769 37836232

[37] Bilali HE, Rokka S, Calabrese G, Borelli T. Conservation and promotion of neglected and underutilized crop species in West Africa: policy and governance. Sustainability. 2024.

[38] Padulosi S, Thompson J, Rudebjer P. Fighting poverty, hunger and malnutrition with neglected and underutilized species (NUS). 2013.

[39] Bankole M, Bodjrènou S, Honfo F, Codo G, Bodecker J, Termote C, et al. Valorization of Vigna radiata (L.) Wilczek and Moringa oleifera to improve food recipes of 6-23-month-old children in northern Benin. J Agric Food Res. 2023;13:100639.

[40] Anggraeni BM, Rahmawaty S. Acceptance of brownies suweg (Amorphophallus paeoniifolius) substituted with mung bean (Vigna radiata). In: Proceedings of the International Conference on Health and Wellness. 2022: 59–64. https://www.atlantis-press.com/proceedings/ichwb-21/125972789

[41] Senyolo GM, Wale E, Ortmann GF. Consumers’ willingness-to-pay for underutilized vegetable crops: the case of African leafy vegetables in South Africa. J Human Ecol. 2014;47(3):219–27. doi: 10.1080/09709274.2014.11906756

[42] Sequeros T, Ochieng J, Schreinemachers P, Binagwa PH, Huelgas ZM, Hapsari RT, et al. Mungbean in Southeast Asia and East Africa: varieties, practices and constraints. Agric Food Secur. 2021;10(1). doi: 10.1186/s40066-020-00273-7

[43] Assogbadjo AE, Glèlè Kakaï R, Chadare FJ, Thomson L, Kyndt T, Sinsin B. Folk classification, perception, and preferences of baobab products in West Africa: consequences for species conservation and improvement. Econ Bot. 2008;62(1):74–84.

[44] Hunter D, Borelli T, Beltrame DMO, Oliveira CNS, Coradin L, Wasike VW, et al. The potential of neglected and underutilized species for improving diets and nutrition. Planta. 2019;250(3):709–29. doi: 10.1007/s00425-019-03169-4 31025196

[45] Mayes S, Massawe FJ, Alderson PG, Roberts JA, Azam-Ali SN, Hermann M. The potential for underutilized crops to improve security of food production. J Exp Bot. 2012;63(3):1075–9. doi: 10.1093/jxb/err396 22131158

[46] Bharucha Z, Pretty J. The roles and values of wild foods in agricultural systems. Phil Trans R Soc B. 2010;365(1554):2913–26. 20713393 10.1098/rstb.2010.0123 PMC2935111

[47] Chadare FJ, Hounhouigan JD, Linnemann AR, Nout MJR, Van Boekel MAJS. Indigenous knowledge and processing of *Adansonia Digitata* L. food products in Benin. Ecol Food Nutrit. 2008;47(4):338–62.

[48] Achigan-Dako EG, Pasquini MW, Assogba Komlan F, N’danikou S, Yédomonhan H, Dansi A. Traditional vegetables in Benin. Cotonou: Institut National des Recherches Agricoles du Bénin; 2010.

[49] Codjia JTC, Assogbadjo AE, Ekué MRM. Diversité et valorisation au niveau local des ressources végétales forestières alimentaires du Bénin. Cahiers Agric. 2003;12(5):321–31.

[50] Bosman MJC, Kruger A, Matenge STP, Van der Merwe M, De Beer H. Consumers’ beliefs on indigenous and traditional foods and acceptance of products made with cow pea leaves. 2012. https://repository.nwu.ac.za/handle/10394/9274

[51] Buchmann C, Prehsler S, Hartl A, Vogl CR. The importance of baobab (*Adansonia digitata* L.) in rural West African subsistence--suggestion of a cautionary approach to international market export of baobab fruits. Ecol Food Nutr. 2010;49(3):145–72. doi: 10.1080/03670241003766014 21883078

[52] Shackleton CM, Pasquini MW, Drescher AW. African indigenous vegetables in urban agriculture: recurring themes and policy lessons for the future. African Indigenous Vegetables in urban agriculture. 2009.

[53] Dossoukpevi R, Ahanhanzo C, Adoukonou-Sagbadja H, Cacai G, Naitchede H, Agbangla C. Contribution à l’amélioration de la production in vitro de deux espèces d’Ocimum spp (Lamiaceae): Ocimum basilicum et Ocimum gratissimum cultivées au Bénin. Int J Biol Chem Sci. 2012;6(6):4046–57.

[54] Baco MN. Ethno-ecological variability in the consumption of leafy green plants in the Republic of Benin. AJAEES. 2019:1–15.

[55] Batawila K, Akpavi S, Wala K, Kanda M. Diversité et gestion des légumes de cueillette au Togo. Developing African leafy vegetables for improved nutrition. 2005: 55–55.

[56] Borelli T, Hunter D, Padulosi S, Amaya N, Meldrum G, de Oliveira Beltrame DM, et al. Local solutions for sustainable food systems: the contribution of orphan crops and wild edible species. Agronomy. 2020;10(2):231. doi: 10.3390/agronomy10020231

[57] Keatinge JDH, Easdown WJ, Yang RY, Chadha ML, Shanmugasundaram S. Overcoming chronic malnutrition in a future warming world: the key importance of mungbean and vegetable soybean. Euphytica. 2011;180(1):129–41. doi: 10.1007/s10681-011-0401-6

[58] Alaofè H, Zhu M, Burney J, Naylor R, Douglas T. Association between women’s empowerment and maternal and child nutrition in Kalalé District of Northern Benin. Food Nutr Bull. 2017;38(3):302–18. doi: 10.1177/0379572117704318 28443373

